# Interobserver agreement for neonatal seizure detection using multichannel EEG

**DOI:** 10.1002/acn3.249

**Published:** 2015-10-01

**Authors:** Nathan J. Stevenson, Robert R. Clancy, Sampsa Vanhatalo, Ingmar Rosén, Janet M. Rennie, Geraldine B. Boylan

**Affiliations:** ^1^ Neonatal Brain Research GroupIrish Centre for Fetal and Neonatal Translational ResearchUniversity College CorkCorkIreland; ^2^ Division of NeurologyThe Children's Hospital of PhiladelphiaPhiladelphiaPennsylvania; ^3^ Departments of Neurology and PediatricsPerelman School of MedicineUniversity of PennsylvaniaPhiladelphiaPennsylvania; ^4^ Department of Clinical NeurophysiologyHUS Medical Imaging CenterHelsinki University Central Hospital and University of HelsinkiHelsinkiFinland; ^5^ Department of Clinical NeurophysiologyLund University HospitalLundSweden; ^6^ Academic Research Department of NeonatologyInstitute for Women's HealthUniversity College LondonLondonUnited Kingdom

## Abstract

**Objective:**

To determine the interobserver agreement (IOA) of neonatal seizure detection using the gold standard of conventional, multichannel EEG.

**Methods:**

A cohort of full‐term neonates at risk of acute encephalopathy was included in this prospective study. The EEG recordings of these neonates were independently reviewed for seizures by three international experts. The IOA was estimated using statistical measures including Fleiss' kappa and percentage agreement assessed over seizure events (event basis) and seizure duration (temporal basis).

**Results:**

A total of 4066 h of EEG recordings from 70 neonates were reviewed with an average of 2555 seizures detected. The IOA was high with temporal assessment resulting in a kappa of 0.827 (95% CI: 0.769–0.865; *n* = 70). The median agreement was 83.0% (interquartile range [IQR]: 76.6–89.5%; *n* = 33) for seizure and 99.7% (IQR: 98.9–99.8%; *n* = 70) for nonseizure EEG. Analysis of events showed a median agreement of 83.0% (IQR: 72.9–86.6%; *n* = 33) for seizures with 0.018 disagreements per hour (IQR: 0.000–0.090 per hour; *n* = 70). Observers were more likely to disagree when a seizure was less than 30 sec. Overall, 33 neonates were diagnosed with seizures and 28 neonates were not, by all three observers. Of the remaining nine neonates with contradictory EEG detections, seven presented with low total seizure burden.

**Interpretation:**

The IOA is high among experts for the detection of neonatal seizures using conventional, multichannel EEG. Agreement is reduced when seizures are rare or have short duration. These findings support EEG‐based decision making in the neonatal intensive care unit, inform EEG interpretation guidelines, and provide benchmarks for seizure detection algorithms.

## Introduction

Neonatal seizures are a medical emergency and arise in 1–5/1000 neonates.^1^ Seizure onset is primarily in the first week of life. Although seizures are intermittent events, status epilepticus is common and the number and duration of seizures varies significantly both within and across etiologies.^2^, ^3^ The total seizure burden in neonates is often very high and seizures may occur repeatedly over a number of days. Antiseizure drug (ASD) treatment is warranted but to optimize the therapy, prompt and accurate seizure identification is required.

The current gold standard for seizure detection is the visual interpretation of conventional multichannel EEG by the human expert.^4^, ^5^ EEG seizures are traditionally characterized by the appearance of sudden, repetitive, evolving, and stereotyped waveforms that have a definite beginning, middle, and end and last for at least 10 sec.^6^ While visual interpretation of the multichannel EEG by human experts outperforms other seizure detection techniques such as bedside clinical assessment^7^ and amplitude‐integrated EEG (aEEG),^8^ information on its reliability is limited. It is imperative that the reliability of the gold standard is known, as it often provides the basis for timely clinical treatment and follow‐up of treatment response with ASD as well as the quantifiable measure of choice in therapeutic trials. An assessment of the reliability of EEG interpretation provides essential support for studies on neonatal seizures, developing guidelines for EEG interpretation and benchmarking computer‐based seizure detection. As EEG interpretation is visual and hence subjective, the best method for assessing its reliability is the interobserver agreement (IOA). There have been several studies that have reported estimates of IOA, however, they compared EEG readers with varying levels of experience, different measures of IOA and limited datasets.^9^, ^10^, ^11^

The aim of this study is to provide a comprehensive assessment of IOA in neonatal seizure detection by visual interpretation of long duration recordings of multichannel EEG using the annotations of three experienced reviewers.

## Method

### Patient population

We used long duration unedited multichannel EEG recordings reflecting the real world clinical scenario. A cohort of neonates, at high risk of seizures, was enrolled for EEG monitoring from the neonatal intensive care units (NICUs) of the Cork University Maternity Hospital, Ireland and the University College London Hospital, UK from January 2009 to October 2011. Neonates were considered to be at high risk of acute encephalopathy if they fulfilled at least one of the following criteria: Apgar score less than six at 5 min; a continued need for resuscitation after birth; and any clinical evidence of encephalopathy including seizures within 72 h of age. The time of any ASD administration was also recorded for each neonate.

### Data acquisition

Multichannel conventional video EEG recording was commenced on enrollment (as soon as possible after birth or presentation with a clinical seizure) and continued for up to 120 h. The EEG recordings were supplemented with electrocardiogram, respiration, and aEEG trend display. A Nicolet One EEG machine (Natus Medical Inc., Pleasanton, CA) was used for these polygraphic recordings. An array of 10–12 scalp electrodes was placed according to the International 10–20 system, modified for neonates, including frontal (F3 and F4), central (C3, C4, and Cz), temporal (T3 and T4), occipital (O1 and O2), and a reference. Electrode to scalp impedance was maintained below 5 kΩ when possible. Neonates were included if the EEG recording length was greater than 12 h.

All the EEGs were recorded with a study identification number and stored without identifiable patient information. Approval for the study was obtained from the Clinical Research Ethics Committees of the Cork Teaching hospitals, Ireland and the Integrated Research Application Service of the National Health Service in the United Kingdom. Written, informed consent was received from at least one parent of each neonate included in the study.

### Seizure annotation

The database of EEG recordings was reviewed by three EEG experts who have trained and practiced at different institutions (I. R., R. C., and S. V.; referred to as observers in the remaining text). Observers were asked to annotate the beginning and end of each electrographic seizure collectively for all EEG channels, not on a region‐by‐region basis. The minimum seizure duration was defined as 10 sec.^6^

Observers were allowed to use any montage, filter setting, and display time base deemed necessary for optimal interpretation. All montages, however, had to include electrodes F3, F4, C3, C4, Cz, T3, T4, O1, and O2. The amount of time required for observers to review and annotate each tracing was recorded. Observers were encouraged to minimize any effects of fatigue by limiting each review session to 2 h with a break of at least 1 h between sessions. They did not have access to the video or clinical notes related to the neonates and were blinded to each other's annotations. The annotations were exported in an ASCII file format with a temporal resolution of 1 sec.

### Interobserver agreement

The measurement of IOA is not a trivial task and must be performed with a battery of complementary measures and data configurations.^5^ Our aim was to determine the IOA between three observers and to uncover the factors that may conceivably affect the IOA. Factors included in analyses were the total number of seizures, their durations, short‐ and long‐term measures of seizure accumulation, and ASD administration. We also quantified the IOA with respect to the underlying etiology of neonatal seizures.

IOAs of the annotations were measured using several temporal and event‐based measures. To avoid limitations in individual measures, such as the sensitivity of kappa to bias and prevalence,^12^ we employed a comprehensive array of measures for the assessment of agreement (see Figure 4 in Boylan et al. 2013 5).

**Figure 1:**
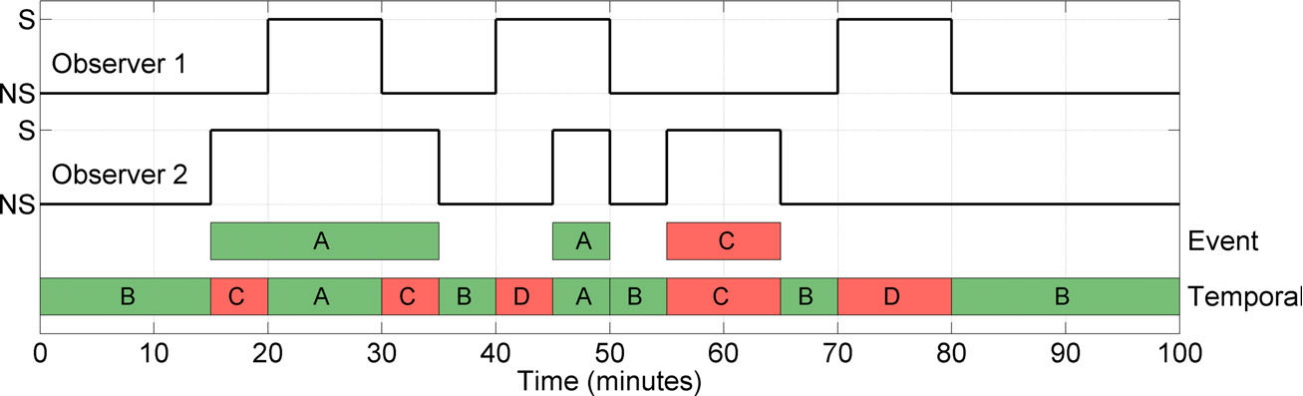
The assessment of agreement between the annotations of two simulated observers. The annotation of observer 2 is compared to observer 1. A – positive (seizure) agreement, B – negative (nonseizure) agreement, C – positive disagreement, D – negative disagreement. In this case, an event‐based assessment results in a positive agreement rate of 66.6% (2/3 events) and 0.6 positive disagreements per hour (one disagreement in 100 min). A temporal assessment results in a positive agreement rate of 50% (15/30 min) and a negative agreement rate of 71.4% (50/70 min). Kappa is calculated using a combination of temporal measures (A–D). These are one‐sided measurements of agreement; in order to form a complete estimate, the average of these measures based on a comparison of observer 1 against observer 2 and observer 2 against observer 1 is used. This averaging is inherent in kappa, which is 0.205, and in this example, equivalent to modified kappa.

The temporal measures include Fleiss' kappa, a modified kappa (to normalize differences in recording duration), positive (seizure) and negative (nonseizure) agreement rate.^13^, ^14^ Event‐based measures include the positive agreement rate and number of positive disagreements per hour.^14^ The estimation of these measures on an example annotation is shown in (Fig. 1). The bias and prevalence index were also estimated to aid interpretation and paired‐observer comparisons were also performed using Cohen's kappa statistic to investigate the possibility of a rogue observer.

The IOA is typically summarized across patients to provide useful information on interpatient variability. The IOA assessment on an individual level, however, requires the presence and absence of seizure in a neonate, which was not the case in approximately half of our neonates. In order to overcome this deficiency, measures were also estimated on recordings that were linked or pooled together (concatenated). This permits the use of all available data and complements summary measures across neonates.

The confidence intervals (CI) were calculated on IOA measures based on the analysis of a concatenated recording using bootstrap resampling (the 95% CI was estimated using 1000 iterations), where a neonate was assumed as a sample. A median and interquartile range (IQR) was used to summarize IOA measures across the cohort of neonates.

The IOA for the identification of any seizure in a neonate and a classification of seizure intensity were also assessed using Fleiss' kappa. Seizure intensity was defined using the peak hourly seizure burden (HSBp). This is a measure of the short‐term accumulation of seizures, as opposed to total seizure burden which is a measurement of the long‐term accumulation of seizures. The HSBp was defined as the maximum accumulated duration of seizures within any hour of the entire EEG recording. It was then used to define three groups with a binary classification of low and high HSBp. Each group was dichotomized with a threshold of: (1) 5 min per hour; (2) 12 min per hour; and (3) 30 min per hour. The first is potentially a minimum burden to initiate treatment with ASDs, the second may be useful for prognosis and the third is a common definition of electrographic status epilepticus.^15^, ^16^, ^17^

The agreement between descriptive measures obtained from the annotations such as the total seizure number, mean and median seizure durations, seizure onset (time of the first seizure), seizure offset (time of the last seizure), and seizure period (seizure offset minus seizure onset) were assessed with the intraclass correlation coefficient (ICC). ICCs were reported with a 95% CI. The effect of seizure duration on positive agreement rate was also examined using a histogram analysis and any association between variables was estimated using Spearman's correlation coefficient.

The effect of ASDs on the IOA was evaluated by comparing the IOA in a period 4 h before ASD administration to a period 4 h after ASD administration. Only doses of ASD that contained seizure before and after ASD administration were included. In this case, the IOA was estimated using kappa/kappa maximum in order to correct for changes in the prevalence due to ASD administration. A Wilcoxon signed‐rank test was used to determine the differences in IOA. A summary of the analysis is shown in (Table 1).

**Table 1:** A summary of the various analyses of interobserver agreement applied to the cohort of 70 neonates

	Kappa	ICC	PA	NA	PD/h
Raw annotations					
Temporal	1,2		1,2	1,3	
Event			1,2		1,3
Interpreted annotations					
Presence of seizure	3				
Presence of SE	3				
Characteristics of seizures		2,3			

Results based on the analysis of the concatenated recording will be summarized as value (95% CI) and results based on the analysis of individual recordings will be summarized across the cohort as median (IQR). ICC, intraclass correlation coefficient; PA, positive (seizure) agreement; NA, negative (nonseizure) agreement; PD/h, positive disagreements per hour.

1, Estimated on concatenated annotations.

2, Estimated on annotations of seizure neonates individually.

3, Estimated on annotations of all neonates individually.

### Sample size calculation

The calculation of sample size assumed a binary outcome of detection of seizure versus no detection of seizure in a neonate. Assuming Fleiss' kappa coefficient of 0.85 for three observers, 70 neonates (35 with seizures and 35 without) would be required in order to achieve a 95% confidence interval of 0.75–0.95. The calculation is based on the asymptotic variance for the Fleiss–Cuzick estimator of kappa.^18^

## Results

A total of 107 neonates met the inclusion criteria for the study. Thirty five neonates were considered to have electrographic seizures based on local interpretations. A database of EEG recordings from 70 neonates was generated for review by the observers, containing recordings from 35 neonates with reported seizures and 35 without, randomly selected from the remaining 72 neonates. A summary of demographics of the cohort of 70 neonates included in this study is shown in (Table 2).

**Table 2:** Demographics and EEG recording information relating to the 70 neonates used in this study

Gestational age (weeks^+days^)^a^	40^+3^ (39^+2^ to 41^+2^)
Birthweight (g)^a^	3526 (3140 to 3920)
Gender (male:female)	37:33
Age at EEG onset (h)^a^	7.0 (3.6 to 19.0)
EEG recording duration (h)^a^	51.6 (21.5 to 84.4)

Primary diagnosis	Neonates (*N*)
HIE^b^	37
Asphyxia^c^	10
Stroke	8
Focal Lesion	3
Other^d^	12

a Median (interquartile range).

b HIE ‐ hypoxic iscaemic encephaloptahy. Ten mild, 19 moderate, eight severe HIE. Twenty‐two were treated with therapeutic hypothermia (TH).

c Asphyxia was defined as depressed Apgar scores with no subsequent signs of encephalopathy.

d Other: includes one or more conditions such as meningitis, viral encephalitis, sepsis, meconium aspiration syndrome, persistent pulmonary hypertension, respiratory distress syndrome, benign familial neonatal seizures, benign sleep myoclonus, and unknown diagnosis.

**Figure 2:**
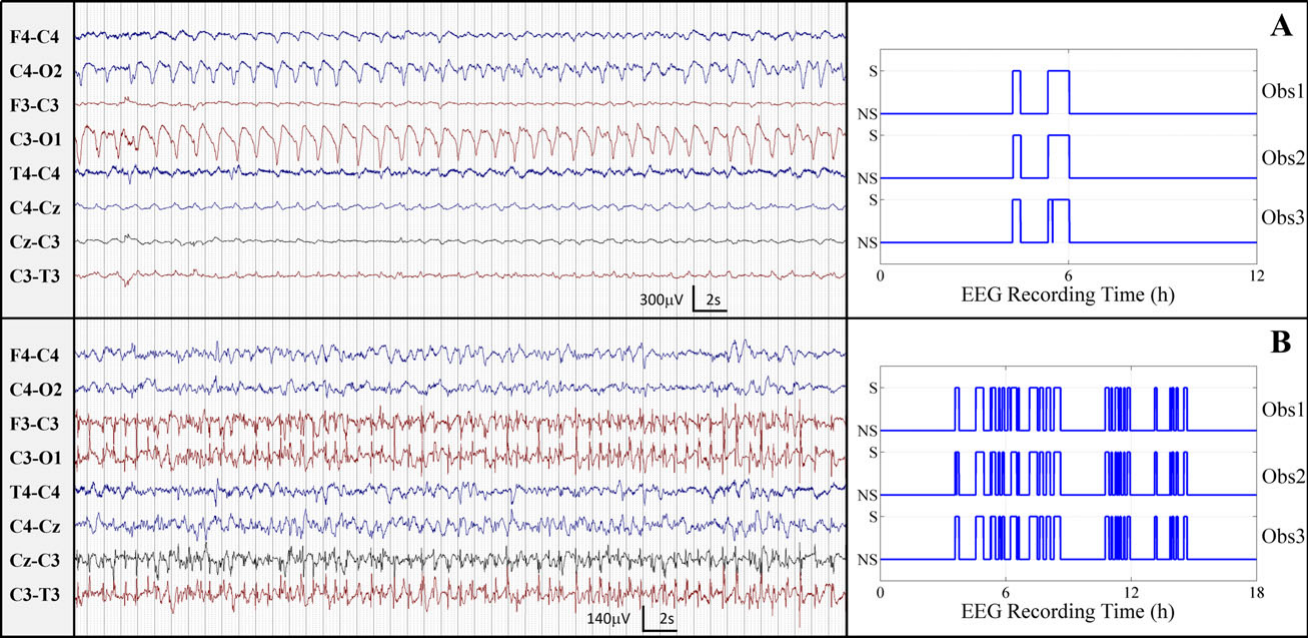
Example seizures from the EEG recordings of neonates with high agreement. (A) kappa was 0.982, total seizure burden was [55, 55, and 56] minutes, and seizure number was [3, 2, and 2], seizure duration = [1106, 1656, and 1675] seconds, for observers 3, 2 and 1 respectively. (B) kappa was 0.950, total seizure burden was [201, 192, and 205] minutes, seizure number was [24, 26, and 24] and seizure duration was [502, 442, and 512] seconds, for observers 1, 2, and 3, respectively. Obs1, Obs2, and Obs3 are the annotations of observer 3, 2, and 1, respectively, S is seizure and NS is nonseizure.

A total of 4066 h of EEG was recorded and reviewed by each observer with an average of 2555 seizures annotated. Examples of EEG seizure and seizure detections are shown in (Fig. 2). The number of neonates who were determined to have had seizure was 37, 35, and 38 for observers 1, 2 and 3, respectively. The approximate time taken to annotate the database of EEG was, on average, 120 h, which corresponds to a review rate of 1.8 min per hour of EEG.

We found high IOA among the three observers. Temporal assessment resulted in a kappa value of 0.827 (95% CI: 0.769–0.865; prevalence 0.952; bias 0.0005). The modified kappa value was 0.836 (95% CI: 0.797–0.868). The positive and negative agreement between the observers was 83.2% (95% CI: 77.3–87.0%) and 99.6% (95% CI: 99.4–99.7%), respectively. The positive agreement rate had a median of 83.0% (IQR: 76.6–89.5%) when summarized across neonates with seizures identified by all three observers (*n* = 33). The negative agreement rate had a median of 99.7% (IQR: 98.9–99.8%) when summarized across all neonates (*n* = 70). Event‐based measures resulted in a seizure agreement rate of 77.9% (95% CI: 66.9–84.7%) and 0.142 (95% CI: 0.083–0.215) false positives per hour. The positive event agreement rate had a median of 83.0% (IQR: 72.9–86.6%) when summarized across neonates with seizure as identified by all three observers (*n* = 33) and a median of 0.018 (IQR: 0.000–0.090) false positives per hour when summarized across all neonates (*n* = 70). There was no rogue observer as the IOA, measured by kappa, was consistent across observers: Obs1 versus Obs2; 0.822 (95% CI: 0.750–0.864), Obs1 versus Obs3; 0.838 (95% CI: 0.794–0.873), and Obs2 versus Obs3; 0.822 (95% CI: 0.755–0.864).

**Figure 3:**
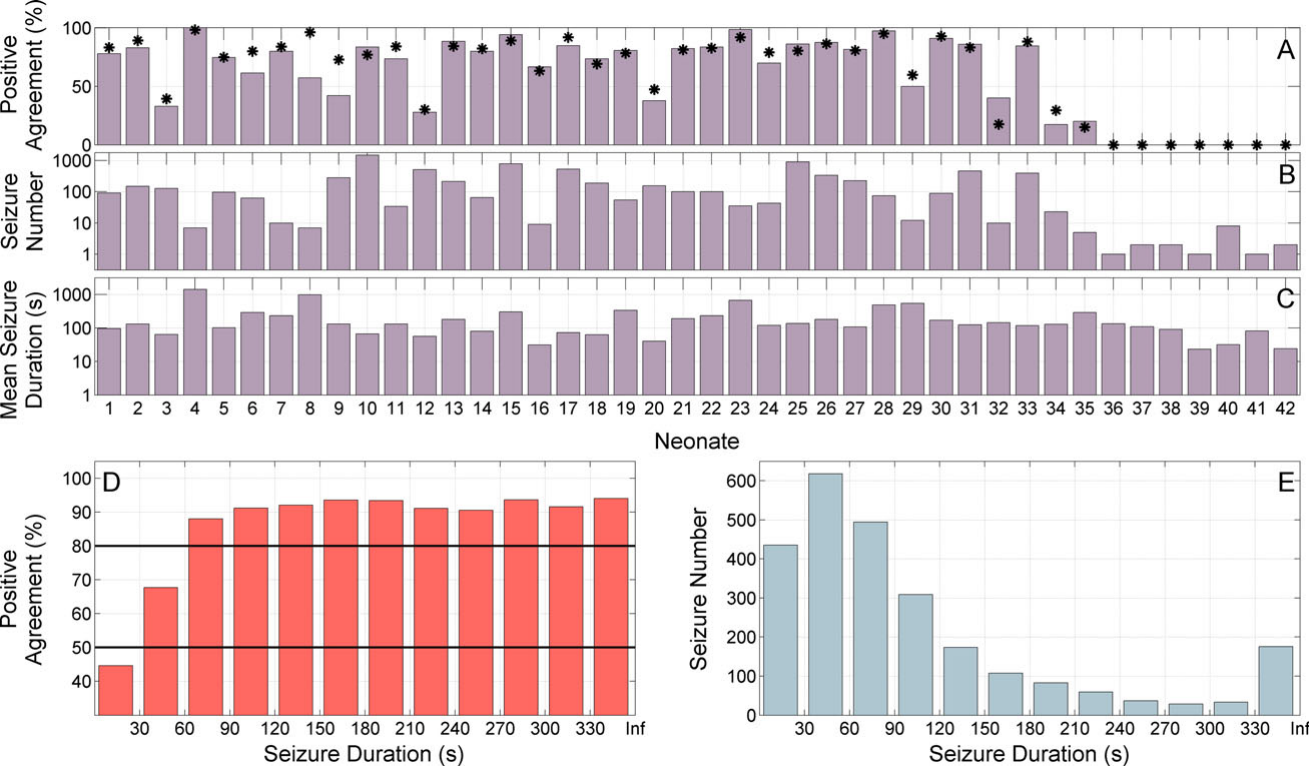
The decrease in interobserver agreement due to seizures with rare occurrence or short duration; in total and on a per neonate basis. (A) The positive agreement (event‐based assessment) per neonate in those with seizures annotated by at least one observer (*n* = 42). The agreement was reduced in neonates with a limited number of seizures, particularly if these seizures were of short duration. The black asterisks are the kappa value (expressed as a percent) for each neonate. Neonates 1–33 had annotations from all 3 observers, neonates 34–35 had annotations from 2 observers (*n* = 2) and neonates 36–42 had annotations from 1 observer (*n* = 7). (B) The number of seizures in each neonate (plotted on a logarithmic scale, base 10). (C) The mean seizure duration in each neonate (plotted on a logarithmic scale, base 10). (D) Positive agreement rate with respect to seizure duration summarized across all seizures. Observers were more likely to disagree if the seizure duration was less than 30 sec. (E) The number of seizures with a given seizure duration. All measures are averaged across observers. For (D and E) the last bin of the histogram contains seizures of duration greater than 330 sec (Inf is infinity).

Some characteristics of seizures affected IOA (Fig. 3A–C). In a subgroup of neonates who had seizures, according to at least one observer (*n* = 42), the average number of positive event agreements was 1978 and the average number of positive event disagreements was 578 (a ratio of 3.4:1). The most common seizure duration was between 30 and 60 sec (Fig. 3E) and observers were more likely to disagree when a seizure was less than 30 sec (Fig. 3D). This level of disagreement in 30 sec seizures was not associated with the underlying total seizure burden of the neonate (*r* = −0.07; 95% CI: −0.27–0.08). The IOA was improved, post hoc, by redesignating seizures less than 30 sec as “nonseizure”: kappa was 0.832 (95% CI: 0.782–0.866), modified kappa was 0.843 (95% CI: 0.804–0.870), event agreement rate was 82.6 (95% CI: 75.1–87.4) at 0.090 (95% CI: 0.050–0.138) disagreements per hour and the ratio of positive event agreement to disagreement was 5.3:1.

We further assessed the effect of redesignating seizures less than 30 sec on the summary EEG judgment and found little change. The number of seizures was reduced by 11%; there was only a negligible reduction on the total seizure burden of each neonate (median reduction of 0.8 min; IQR: 0.0–3.1 min; *n* = 42) and seizure onset was unchanged (median 0 h; IQR: 0–0 h; *n* = 42). The most marked difference was seen in two neonates who were reclassified as having no seizures. These neonates had low total seizure burden (23 and 48 sec) identified by only a single observer.

A total of 97 doses of ASDs were recorded. Of these, 46 doses in 20 neonates were sufficient for analyzing the effect of ASD administration. The IOA was not significantly altered by ASD administration (*P* = 0.65; *n* = 20).

**Figure 4:**
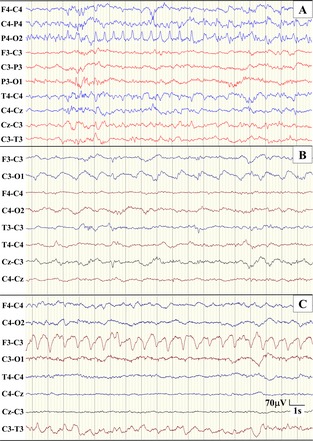
Example EEG where observers disagreed on the presence of seizure. The montage used at the time of recording is used to present the EEGs. (A) Seizure identified by one observer, total seizure burden was 3 min and seizure number was 2 – predominant seizure activity on O2. (B) Seizure identified by two observers, total seizure burden was [23.5, 26.1] minutes and seizure number was [12, 11] – predominant seizure activity on O1. (C) Seizure identified by one observer, total seizure burden was 3.7 min and seizure number was 2 – predominant seizure activity on F3. The scale is identical for all EEG segments.

EEG diagnosis of seizures in a given neonate was highly congruent among observers, with a kappa of 0.828 (95% CI: 0.694–0.923; prevalence 0.048; bias 0.029). The relationship between IOA and the HSBp are summarized in (Table 3). There were 33 neonates where all three observers identified the presence of seizure and 28 neonates where all three observers did not identify any seizures. Of the remaining nine neonates, 7 (78%) were identified as having seizure by only one observer (Table 4). In these neonates, the total seizure burden measured was less than 5 min during the entire period of EEG‐monitoring period (median duration 42.5 h; IQR 17.3–66.3 h). Six had only one or two seizures, while the remaining neonate had eight short‐duration seizures. In the majority of neonates where at least one observer did not identify seizure, the predominant seizure activity was situated on only one channel (Fig. 4). To assess the influence of total seizure burden on IOA we recast the definition of a “seizure‐positive” patient, post hoc, from a total seizure burden greater than 0 min to a total seizure burden greater than 5 min. This changed the binary classification of 13/210 (6%) neonates across the three observers and resulted in an improved kappa of 0.943 (95% CI: 0.865–1.000).

**Table 3:** The relationship between interobserver agreement and the degree of peak hourly seizure burden (HSBp)

	HSBp cutoff		
	>5 mins/h	>12 mins/h	>30 mins/h
Kappa	0.904 (0.809–0.980)	0.818 (0.696–0.920)	0.815 (0.641–0.950)
PI	0.105	0.238	0.619
BI	0.010	0.019	0.010
*N*	34	31	17
3 Observers	29	22	11
2 Observers	2	5	1
1 Observer	3	4	5

Kappa is presented as value (95% CI). PI is the prevalence index, BI is the bias index, N is the number of neonates identified as exceeding the HSBp by at least one observer, and X observers defines the number of observers who determined that the HSBp was exceeded.

**Table 4:** Seizure characteristics in nine neonates where at least one observer did not agree on the presence of seizures

Neonate	Cohen's *κ* a	Observers	TSB (minutes)	Seizure number	Mean seizure duration (seconds)	Channel localization
1	–	3	2.2	1	133	Generalized
2	–	3	3.7	2	110	Focal (P4)
3	–	1	3	2	90	Focal (F3, T3)
4	–	3	0.4	1	23	Focal (F4)
5	–	2	4.3	8	32	Generalized and Focal (P3, T3)
6	–	1	1.4	1	81	Focal (O2)
7	–	1	0.8	2	24	Focal (Cz)
8	0.592	2, 3	23.5, 26.1	12, 11	118, 142	Focal (O1, O2)
9	0.302	1, 3	20.1, 3.7	4, 1	310, 221	Focal (T4, O2)

In the case where two observers identified seizures, the individual characteristics of each observer's annotation have been listed. κ is kappa, TSB is the total seizure burden.

a Estimated when at least two observers have detected seizures in a neonate.

A high level of IOA between observers translates to high IOA in descriptive measures of seizures per neonate (Table 5). Nevertheless, the ICC varies depending on the seizure measure. Importantly in the clinical context, the assessment of total seizure burden was the most reliable measure of neonatal seizures.

**Table 5:** Characteristics of seizures as annotated by each observer summarized across neonates with seizures

	Observer 1 (*n* = 37)	Observer 2 (*n* = 35)	Observer 3 (*n* = 38)	ICC (*n* = 70)
Total seizure burden (mins)	79.3 (25.4–199.8)	87.7 (32.7–197.8)	74.7 (24.6–189.5)	0.996 (0.994–0.997)
Seizure number	24 (6–73)	28 (11–106)	27 (5–77)	0.974 (0.962–0.983)
Seizure duration (mean) (sec)	155 (84–319)	126 (90–253)	128 (90–221)	0.923 (0.885–0.950)
Seizure Duration (median) (sec)	83 (56–140)	93 (56–155)	83 (55–140)	0.866 (0.801–0.913)
Seizure onset (h)	17.2 (10.8–34.6)	17.7 (10.6–35.8)	19.0 (10.9–33.8)	0.982^a^ (0.969–0.991)
Seizure offset (h)	50.8 (32.2–92.1)	50.6 (34.5–100.0)	54.8 (33.9–106.1)	0.912^a^ (0.844–0.954)
Seizure period (h)	20.3 (8.6–40.8)	19.3 (7.6–54.2)	22.8 (10.6–52.3)	0.954^a^ (0.918–0.976)

a Total seizure burden is the accumulated duration of seizure, seizure onset/offset are the age of first/last seizures, respectively and seizure period is the time between first and last seizures in a neonate. Results are reported as median (IQR). ICC, intraclass correlation coefficient. The ICC is the average ICC, is presented as ICC (95% CI) and is estimated across the entire cohort (*n* = 70), unless^1^ at which it is estimated on the 33 neonates where all observers detected at least one seizure.

The etiology of the 33 neonates where seizures were annotated by all three observers was moderate or severe Hypoxic ischaemic encephalopathy (HIE) (*n* = 18), stroke (*n* = 8), focal lesions (*n* = 3), benign familial neonatal seizures (*n* = 2), meningitis (*n* = 1), viral encephalopathy (*n* = 1), and unknown diagnosis (*n* = 1). The diagnosis of two neonates where seizures were annotated by two observers was moderate HIE (*n* = 2). The diagnoses of seven neonates where seizures were annotated by one observer were: mild or moderate HIE (*n* = 4), depressed Apgar scores without encephalopathy (*n* = 2), and unknown diagnosis (*n* = 1). There were 13 neonates with a kappa less than 0.5 of which 6 (46%) were diagnosed with HIE and treated with therapeutic hypothermia. Limitations in the sample sizes of subgroups based on etiology preclude any statistical analysis.

## Discussion

We have shown that IOA between experts is high for the detection of neonatal seizures using long duration recordings of multichannel EEG. This work extends the prior literature by providing a comprehensive assessment of IOA evaluated on an exceptionally large dataset of unselected EEG recorded from a NICU covering a realistic spectrum of etiologies. Furthermore, we consider these findings generalizable as the EEG annotations were performed by three experts who were trained in different countries and eras, unrelated to the hospital where the EEGs were recorded, and blinded to each other's assessments and the patients' videos. The experts were also allowed to use their own choice of EEG reading methods, including the EEG montage, time and amplitude sensitivities, and display colors, which could affect IOA if required by arbitrary study design.

Prior studies of IOA for EEG seizure detection have reported variable results. High levels of agreement, comparable to our study, were reported for the detection of adult seizures,^14^ as well as for short duration recordings from a small cohort of seven neonates with seizures.^11^ In contrast, a recent study on older, acutely ill children with seizures reported a much lower kappa of 0.47,^17^ and another study in short duration recordings from 20 neonates reported an averaged ICC (equivalent to kappa for a binary outcome) of 0.57.^10^ A key difference in study methods is the length of EEG recording used. Short duration “snapshot” EEG recordings are not recommended for critically ill neonates.^5^, ^20^ We used long duration conventional EEG monitoring to assess IOA, which more accurately reflects the contemporary clinical NICU environment and fulfills the recommendations of the American Clinical Neurophysiology Society.^20^ As a result, EEG monitoring may be continued for up to 90 h in full‐term neonates, particularly those requiring therapeutic hypothermia. A side effect of such long recordings, however, is a marked reduction in the proportion of seizure to nonseizure annotations (the prevalence) resulting in a reduction in kappa relative to a kappa estimated on shorter duration recordings where periods of higher seizure burden tend to be targeted, resulting in a more equal prevalence.^12^

Closer inspection of our results supports the conclusion that the reliability of seizure detection depends on the accumulation of visual evidence of seizures in the EEG. We showed that IOA was low for seizures with a duration less than 30 sec. Observers had difficulty in ascertaining ictal evolution, a critical feature of recognition when the seizure was short. The IOA was also reduced when the accumulated duration of all seizures was less than 5 min. Observers were more confident when total seizure (burden) burden was high. Of all the metrics considered to quantify seizure severity, the measure “total seizure burden” showed the highest IOA. This finding is particularly important for studies that need to reliably quantify the response of seizures to ASD treatment.^21^

The IOA of seizure detection using only visual interpretation of the EEG was improved by redefining seizure duration to be greater than 30 sec. The diagnosis of “seizure‐positive” and “seizure‐negative” neonates was also improved when the threshold of total seizure burden used to define the diagnosis was shifted from 0 to 5 min. These findings have implications for neonatal EEG interpretation guidelines as a basic expectation for these guidelines is high diagnostic reliability.

A potential limitation in our work is the method of EEG interpretation, without clinical information or video. While this may be a more realistic scenario in a research setting where large databases of neonatal EEG are reviewed in isolation, in clinical practice, readers may avail themselves to the patient's clinical status and video to support or refute the EEG detection of seizures. Consequently, this study may have underestimated the agreement among clinical readers. The IOA, however, is sufficiently high that any improvements from the use of video and clinical information would be minimal. Furthermore, limitations were placed on the level of detail required from observers with only the start and end times of each seizure annotated in order to facilitate efficient processing of the dataset. More detailed annotations could be used to determine if certain seizure‐specific factors such as channel localization, voltage, frequency, and morphology had an effect on periods of disagreement.

Prolonged cot‐side EEG monitoring is a key component of neonatal neurointensive care,^22^ an approach which is gaining ground in the intensive care nurseries around the world. Accurate interpretation of the EEG is currently the main barrier to more widespread uptake of this approach. Expertise in neonatal neurophysiology is scarce, expensive, and reporting is a time‐consuming task. Our experts reported on over 4000 h of EEG from 70 patients, a task which took them 120 h of effort. Computer based methods of analysis have the potential to provide widely accessible EEG interpretation. The level of agreement reached by the human expert, however, was impressive with a "false positive" rate of one every 7 h of reviewed EEG. A result which, so far, has not been matched by any automated method of neonatal seizure detection at equivalent seizure detection rates.^23^, ^24^, ^25^, ^26^ These results set a target of aspiration for such systems, which are relatively low cost, indefatigable, and constantly available.

## Author Contributions

N. J. S. drafted the manuscript, designed and implemented the analysis of the EEG recordings for interobserver agreement and performed any statistical analyses. I. R., S. V., R. R. C. performed the annotation of the EEG recordings used in the study. They also reviewed and revised the manuscript. J. M. R. reviewed and revised the manuscript. G. B. B. designed the protocol for the EEG recording and annotation, coordinated the study, reviewed and revised the manuscript and obtained funding for the study.

## Conflict of Interest

None declared.
